# Overexpression of the *PeaT1* Elicitor Gene from *Alternaria tenuissima* Improves Drought Tolerance in Rice Plants *via* Interaction with a Myo-Inositol Oxygenase

**DOI:** 10.3389/fpls.2017.00970

**Published:** 2017-06-09

**Authors:** Fachao Shi, Yijie Dong, Yi Zhang, Xiufeng Yang, Dewen Qiu

**Affiliations:** ^1^Key Laboratory for Biological Control of the Ministry of Agriculture, Institute of Plant Protection, Chinese Academy of Agricultural SciencesBeijing, China; ^2^State Key Laboratory of Agrobiotechnology, China Agricultural UniversityBeijing, China

**Keywords:** elicitor, PeaT1, interaction, *OsMIOX*, overexpression

## Abstract

Abiotic stresses, especially drought, seriously threaten cereal crops yields and quality. In this study, we observed that the rice plants of overexpression the *Alternariatenuissima PeaT1* gene showed enhanced drought stress tolerance and increased the survival rate following a drought treatment. In *PeaT1*-overexpressing (PeaT1OE) plants, abscisic acid and chlorophyll content significantly increased, while the malondialdehyde (MDA) content decreased compared with the wild-type plants. Additionally, we confirmed that the transcript levels of drought-responsive genes, including *OsAM1*, *OsLP2*, and *OsDST*, were prominently lower in the PeaT1OE plants. In contrast, expression levels of genes encoding positive drought stress regulators including *OsSKIPa*, *OsCPK9, OsNAC9, OSEREBP1*, and *OsTPKb* were upregulated in PeaT1OE plants. Furthermore, combing the yeast two-hybrid assay, we found that PeaT1 could interact with amyo-inositol oxygenase (OsMIOX), which was verified by pull-down assay. Interestingly, *OsMIOX* was highly expressed in PeaT1OE plants during the drought treatment. Additionally, the OsMIOX-GFP fusion protein co-localized with the endoplasmic reticulum (ER) marker in tobacco protoplasts, suggesting OsMIOX performs its function in ER. Therefore, our results are useful for elucidating the molecular mechanism underlying the improvement of drought tolerance by PeaT1.

## Introduction

Drought is one of main abiotic stresses that negatively influences plant development, growth, and seed production (Huang et al., 2009). Plants have evolved diverse mechanisms to respond to environmental stresses. Therefore, details regarding to the molecular mechanism regulating plant responses to stresses may be useful for improving plant resistance to abiotic stresses, especially water deficiency (Huang et al., 2009). Previous studies on *Arabidopsis thaliana* and rice revealed that complex plant responses to drought stress are mediated by several factors, including abscisic acid (ABA) (Ma et al., 2009; Park et al., 2009), reactive oxygen species (Pei et al., 2000; Kwak et al., 2003), and transcription factors (Huang et al., 2009; Fujita et al., 2011). However, biological functions of well known stress-responsive genes remain uncharacterized. Additionally, there are likely many plant genes associated with drought tolerance yet to be identified.

Abscisic acid, which is an important plant hormone, mediates some aspects of the plant life cycle such as seed dormancy, flowering, and fruit ripening. It is also crucial for plant responses to diverse abiotic stresses such as drought, cold, and salinity. Furthermore, ABA regulates stomatal closure to help plants adapt to water deficiency (Zhu, 2002; Fan et al., 2004). In response to abiotic stresses, ABA content immediately increases to protect plants from damages. When plants face with drought stress, ABA signal pathway becomes active and involves in ABA receptors PYR/PYL/RCAR (Park et al., 2009) as well as two phosphatase/kinase enzyme pairs, PP2Cs (Nishimura et al., 2009) and SnRK2s (Gonzalez-Guzman et al., 2012), with opposite functions. In the end, a number of transcription factors under the control of SnRK2s activate ABA dependent genes expression such as ABI5 and ABRE binding factors/ABRE binding proteins (ABF/AREBs), which specifically bind to promoters containing ABA-responsive elements (ABREs) (Antoni et al., 2011), and regulate guard cell channel activities (Lee et al., 2013), resulting in drought-resistant responses (Kim, 2014).

Elicitors isolated from several fungal and bacterial pathogens include proteins, peptides, glycoproteins, lipids, and oligosaccharides (Nürnberger, 1999). Elicitors, which function as signaling molecules, were originally identified in studies that focused on host specificity and disease development (Paré et al., 2005). Harpin, the first reported elicitor from the bacterium *Erwinia amylovora* (Wei et al., 1992; He et al., 1993), could activate the hypersensitive response and growth systems in several crops. The PeaT1 elicitor isolated from *Alternaria tenuissima* induces the systemic accumulation of the pathogenesis-related proteins PR-1a and PR-1b, which are markers for systemic acquired resistance (Zhang W. et al., 2011). Elicitors may also influence plant resistance to environmental stresses, especially drought. For example, PebC1, which was isolated from *Botrytis cinerea*, induces plant drought tolerance (Zhang et al., 2010). Other studies have revealed that PeaT1 could promote wheat and cotton growth and development (Wang et al., 2011; Tang et al., 2012). Additionally, rice and wheat leaves sprayed with exogenous PeaT1 were observed to exhibit increased drought resistance (Liu et al., 2009; Wang et al., 2011). Furthermore, overexpression Harpin-encoding *hrf1* gene in rice enhances drought tolerance (Zhang L. et al., 2011).

Myo-inositol oxygenase (MIOX) is considered as a unique monooxygenase that catalyzes the transfer of myo-inositol to D-glucuronic acid (D-GlcUA), which is an important sugar precursor for plant cell walls (Kanter et al., 2005). Myo-inositol oxygenase balances the myo-inositol content and is essential for the synthesis of some low molecular weight compounds in plants (Smirnoff et al., 2001). Recently, *OsMIOX* expression was observed to be induced by drought, H_2_O_2_, salt, cold, and ABA. Additionally, overexpression the *OsMIOX* considerably improves plant growth under mannitol stress conditions (Duan et al., 2012).

Our lab previously generated PeaT1-overexpressing rice plants (PeaT1OE) (Sheng et al., 2011). In the current study, we analyzed the resistance of PeaT1OE plants to drought stress and using yeast two-hybrid and pull-down assays, found that PeaT1surely interacts with OsMIOX. Thus, our resultss might clarify PeaT1effects on drought tolerance regarding to survival rate, ABA content, and phenotype.

## Materials and Methods

### Drought Tolerance Analysis

In our previous study, an elicitor peaT1 gene was introduced into pCAMBIA2300 vector under the control of CaMV 35S promoter and then transformed into the Nipponbare rice (Sheng et al., 2011). Drought tolerance of transgenic plants was examined by germinating T_2_ transgenic plants. Seeds were submerged in water at room temperature for 48 h, and then allowed to germinate for 48 h at 37°C in a growth chamber. After 4 days, seedlings were transplanted to containers filled with soil, and then incubated in a greenhouse at 24–30°C and 50–60% relative humidity. Wild-type (WT) *Oryza sativa* L. *japonica* cv Nipponbare seedlings and the transgenic seedlings were on soil with three control lines and the PeaT1OE1, PeaT1OE40, and PeaT1OE43 lines, which contained the Nipponbare background. Each line consisted of 10 seedlings. Approximately 21 days later, watering of the seedlings was stopped for 12 days to simulate drought conditions. Seedling survival rates were recorded 15 days after watering was resumed. The drought stress experiment was conducted at least three times.

### Chlorophyll, ABA, Malondialdehyde and Proline Measurements

The chlorophyll extracted from 50 mg (fresh weight) leaf tissue was quantified using the absorbance values at 652 nm according to the Infinite M200 multimode reader (Tecan Group Ltd, Switzerland) as previously described by Kong et al. (2006). ABA was extracted from seedling leaves and quantified according to a previously described method (Fu et al., 2012). Chlorophyll and ABA contents were measured before and during the simulated drought treatment. For every line (10 seedlings), a total of 50 mg tissue was collected from three leaves. Malondialdehyde (MDA) content was measured using a published procedure (Heath and Packer, 1968). Proline concentration was determined as described (Bates et al., 1973). All measurements contained three independent biological replicates. Significant differences are determined with Student’s *t*-test (^∗^0.01 < *P* < 0.05, ^∗∗^*P* < 0.01).

### Quantitative Real-Time PCR Analysis

Total RNA was extracted from WT and PeaT1OE transgenic rice plants using Trizol reagent (TaKaRa, Japan) according to the procedure recommended by the manufacturer. The first-strand cDNA was synthesized in a 20-μl solution using the QuantiTect Reverse Transcription Kit (Qiagen, Germany). The 20 μl qRT-PCR solutions included of 0.5 μl cDNA, 0.2 μM primer mix, and reagents from the SYBR Premix Ex Taq Kit (TaKaRa, Japan). The qRT-PCR was conducted using the ABI PRISM 7900HT system (Applied Biosystems, United States) (Livak and Schmittgen, 2001), with the endogenous rice gene *Ubiquitin* (*LOC_Os03g13170*) serving as the control. Semi quantitative RT-PCR was determined according to method (Marone et al., 2001). qRT-PCR was performed with three technical and three biological replicates. All qRT-PCR primers are listed in Supplementary Table S1. Significant differences are determined with Student’s *t*-test (^∗^0.01 < *P* < 0.05, ^∗∗^*P* < 0.01).

### Drought-Responsive Genes Expression

Drought-responsive genes were detected by quantitative real-time polymerase chain reaction (qRT-PCR) analysis. Water was withheld from 4-week-old rice seedlings for 12 days, after which transcript levels of drought-responsive genes in leaves were analyzed (*OsAM1*, *OsLP2*, *OsDST*, *OsCPK9*, *OsSKIPa OsNCED2, OsNAC9*, *OsTPKb*, and *OsEREBP1*). Additionally, *OsMIOX* expression was detected by exposing 4-week-old transgenic seedlings to air (without water), and then collecting leaves 0.5, 1, 2, 3, 5, 7, and 9 h later for a subsequent qRT-PCR, qRT-PCR was performed with three technical and three biological replicates. All primers are listed in Supplementary Table S1.

### Yeast Two-Hybrid Assay

The *PeaT1* and *OsMIOX* coding sequences were amplified using gene-specific primer sets (Supplementary Table S1). The full-length *PeaT1* sequence was ligated into the pDBleu vector to form the BD-PeaT1 plasmid. Meanwhile, the full-length *OsMIOX* sequence was cloned into the pPC86 vector to generate the AD-OsMIOX plasmid. Yeast cells were transformed and the subsequent reporter gene assays were completed according to the manufacturer’s instructions (Invitrogen, United States).

### Pull-Down Assay

For the *in vitro* pull-down assay, the *OsMIOX* and *PeaT1* coding regions were inserted into pMAL-c2X and pGEX6p-1 vector, respectively, to generate the OsMIOX–maltose-binding protein (MBP) and PeaT1-glutathione *S*-transferase (GST) plasmids (Supplementary Table S1 for primer sets). The amylose resin for purifying MBP (New England Biolabs) and the GST-binding resin for purifying GST (Merck, Germany) were used to purify the fusion proteins and empty tags. The pull-down assay was conducted as previously described (Zhou et al., 2013). Protein products were detected using an enhanced chemiluminescence reagent (GE Healthcare, China).

### Subcellular Localization of OsMIOX

To prepare the pCAMBIA1305-d35S-MIOX-green fluorescent protein (GFP) plasmid, the *MIOX* coding sequence was cloned into the *Bgl* II site of the pCAMBIA1305-GFP vector. The construct was transiently expressed in tobacco (*Nicotiana benthamiana*) epidermal cells as previously described (Batoko et al., 2000).

## Results

### Enhanced Drought Tolerance of PeaT1OE Rice Plants

**FIGURE 1:**
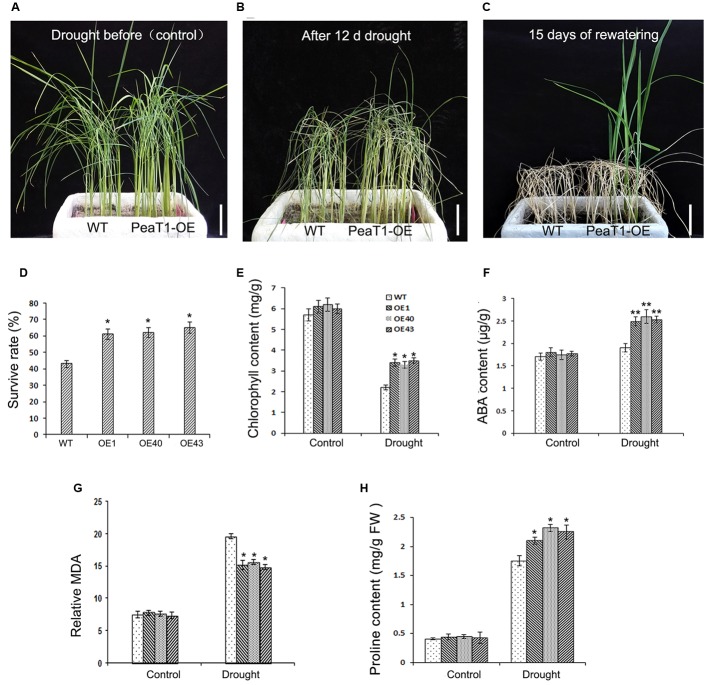
Drought stress tolerance in wild-type (WT) and PeaT1OE lines. **(A)** Three-week-old seedlings. **(B)** Phenotypes of plants deprived of water for 12 days. **(C)** Phenotypes of plants 15 days after watering was resumed. **(D)** Chlorophyll contents of control and PeaT1OE plants, with or without the drought treatment. **(E)** Survival rates were calculated 15 days after watering was resumed. **(F)** Abscisic acid (ABA) contents of the control and PeaT1OE plants, with or without the drought treatment. **(G)** Malondialdehyde (MDA) contents of the control and PeaT1OE plants, with or without the drought treatment. **(H)** Proline contents of control and PeaT1OE plants, with or without the drought treatment. ^∗^0.01 < *P* < 0.05, ^∗∗^*P* < 0.01; Bar in **(A–C)** is 5 cm.

To investigate PeaT1OE activities in drought-stressed plants, 3-week-old WT and PeaT1OE seedlings (**Figure 1A**) were subjected to simulated drought conditions for 12 days (**Figure 1B**). Control leaves demonstrated curled 2 days earlier than PeaT1OE leaves. Additionally, three PeaT1OE lines exhibited leaf wilting and rolling symptoms after the 12-day drought treatment. Fifteen days after watering was resumed (Figure1C), 60% of PeaT1OE plants were still alive, whereas the survival rate of the control plants was only 40% (**Figure 1D**).

Chlorophyll and ABA contents are used to determine whether plants have been exposed to drought stress (Seki et al., 2007; Li et al., 2013). In the current study, we determined that there was no difference in the chlorophyll content between WT and PeaT1OE plants grown under normal conditions. In contrast, among plants exposed to simulated drought conditions, the chlorophyll content of PeaT1OE lines was 50% higher than that of WT plants (**Figure 1E**). Leaf ABA content is correlated with adaptations to environmental stresses via a feedback loop. Thus, we quantified the ABA contents of PeaT1OE and WT leaves under normal and drought conditions. Results showed that there were no significant differences between the two lines before the drought treatment (**Figure 1F**). However, under drought stress, ABA content increased by 38 and 11% in the PeaT1OE and WT plants, respectively.

Malondialdehyde is the end-product of the peroxidation of membrane lipids. It is a consequence of oxidative damage derived from the decomposition of polyunsaturated fatty acid hydroperoxides. A comparison of the MDA contents of WT and PeaT1OE plants revealed that no obvious differences were detected before the drought treatment. However, in response to drought conditions, MDA content decreased significantly (0.01 < *P* < 0.05) in PeaT1OE plants. The result indicated that minor lipid peroxidation occurring in PeaT1OE plants contributed to drought tolerance.

Proline is associated with adaptation to stress abiotic stress in plant (Jiang et al., 2016). We also checked the content of proline in WT and PeaT1OE plant before and during the drought treatment. Nonetheless, no differences were observed under normal conditions (**Figure 1H**). Proline content improved to higher level in PeaT1OE plants, whereas WT plants showed a low increase in proline. These changes demonstrate that proline accumulation are corresponded to the increased drought tolerance of PeaT1OE plants.

### Analysis of Drought-Responsive Gene Expression Levels

**FIGURE 2:**
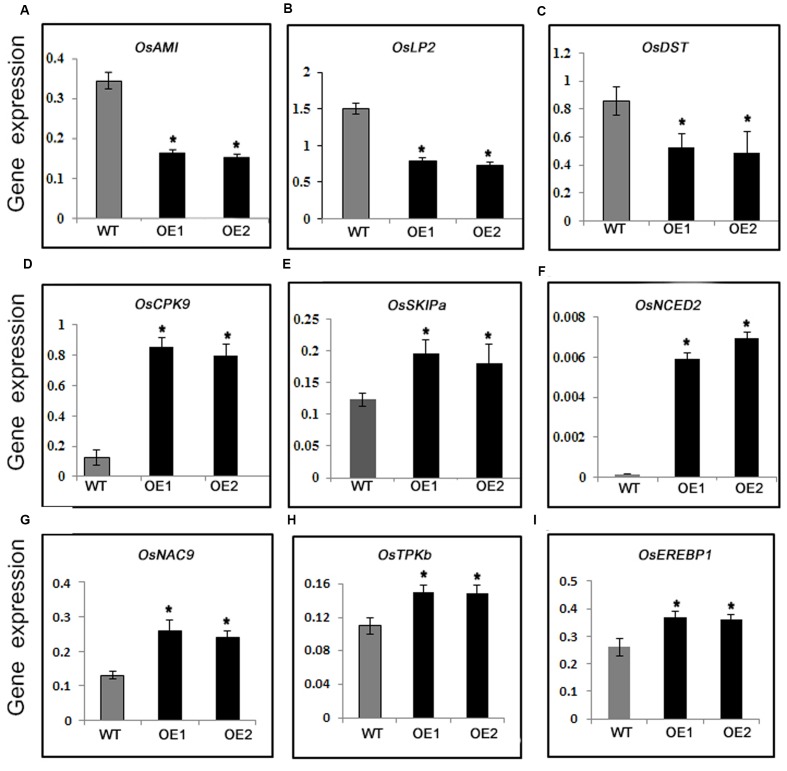
Relative expression levels of drought-responsive genes in WT and PeaT1OE plants. The analyzed genes were *OsAM1* **(A)**, *OsLP2* **(B)**, *OsDST* **(C)**, *OsCPK9* **(D)**, *OsSKIPa* **(E)**, and *OsNCED2* **(F)**, *OsNAC9* **(G)**, *OSTPKb* **(H)**, *OsEREBP1* **(I)**. Data is presented as the mean ± standard deviation (*n* = 9). Significant differences are determined with Student’s *t*-test (^∗^0.01 < *P* < 0.05, ^∗∗^*P* < 0.01).

For a more thorough characterization of the mechanism regulating drought tolerance in PeaT1OE plants, expression levels of several stress-responsive genes were investigated. *OsAM1* gene encodes a K^+^ efflux antiporter that is mainly present in plastid-containing organisms, from lower green algae to higher flowering plants. The expression of *OsAM1* is reportedly induced by polyethylene glycol and salt. Additionally, an *am1* mutant was observed to be more drought tolerant than WT plants (Sheng et al., 2014). In this study, the *OsAM1* transcript level decreased significantly in PeaT1OE plants (**Figure 2A**), suggesting decreased abundance of K^+^ efflux antiporters may be associated with increased drought tolerance of PeaT1OE plants. Furthermore, *OsLP2* (Leaf Panicle 2) (Sheng et al., 2014; Wu et al., 2014) and *OsDST* (Huang et al., 2009) expression levels were much lower in PeaT1OE lines than in WT (**Figures 2B,C**). Additionally, *OsCPK9* encodes a calcium-dependent protein kinase that affects abiotic stress tolerance (Wei et al., 2014). This gene was highly expressed in PeaT1OE plants during the exposure to drought conditions (**Figure 2D**). The transcript level of *OsSKIPa*, which encodes a rice homolog of the human Ski-interacting protein, was higher in PeaT1OE plants than in WT (**Figure 2E**), indicating the expression of drought-responsive genes changed much a lot in PeaT1OE plants.

Abscisic acid is crucial for plant drought stress responses, especially the closure of stomata (Lim et al., 2015). Therefore, we analyzed the expression of *OsNCED2* (9-*cis*-epoxycarotenoid dioxygenase), which is important for ABA biosynthesis. The *OsNCED2*expression level was higher in the PeaT1OE plants than in the WT plants (**Figure 2F**), suggesting that increased ABA biosynthesis may regulate drought resistance in rice plants.

OsNAC9, a member of rice NAC domain family, had been reported to improve drought tolerance in rice (Redillas et al., 2012). In our study, *OsNAC9* was up-regulated in PeaT1OE plants (**Figure 2G**). *OsTPKb*, which encodes a two pore potassium channel protein (Ahmad et al., 2016), decreased the transpiration in its overexpression rice plants under drought stress. We found that the transcription level of *OsTPKb* was higher in PeaTOE plants (**Figure 2H**). Overexpression of an AP2/ERF transcription factor *OsEREBP1* confers drought tolerance in rice (Jisha et al., 2015). Our detection showed that *OsEREBP1* expression level was higher in PeaT1OE plants than WT (**Figure 2I**). Above all, the regulation of drought inducible genes was contributed in PeaT1OE plants to drought tolerance.

### PeaT1 Interacts with OsMIOX

**FIGURE 3:**
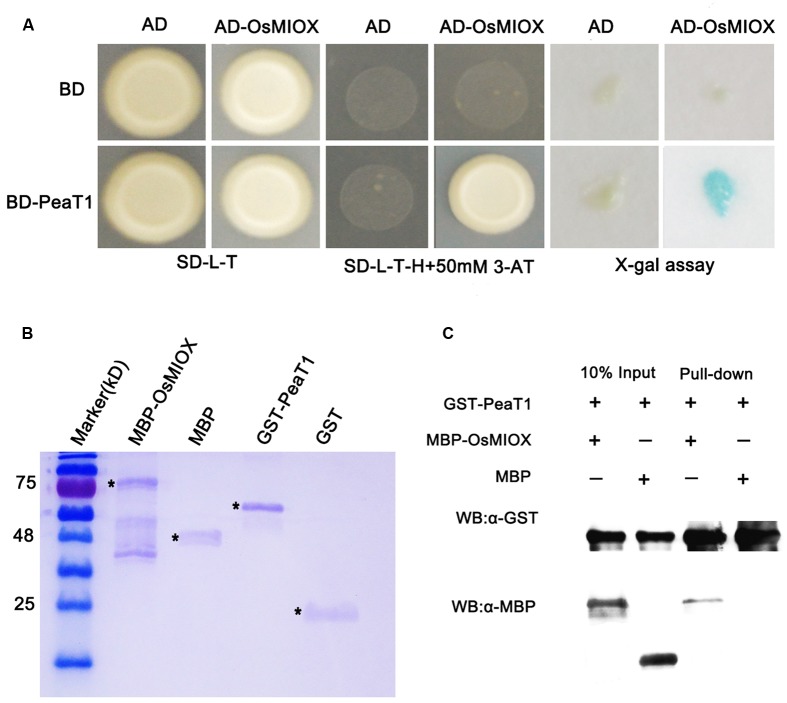
PeaT1 interacts with OsMIOX. **(A)** Results of a yeast two-hybrid assay involving BD-PeaT1 and AD-OsMIOX. The transformed yeast cells were plated on SD/-Leu/-Trp medium (left) and SD/-Leu/-Trp/-His medium supplemented with 50 mM 3-AT (middle). The results of the X-gal assay are also provided (right). **(B)** Purified proteins stained with Coomassie Brilliant Blue. **(C)** A solution consisting of PeaT1-GST bound to the GST-binding resin was incubated with an equal volume of cell lysate containing OsMIOX-MBP or MBP. The binding of OsMIOX-MBP or MBP to the GST-binding resin before (Input) and after (Pull-down) washes was examined in a western blot involving the anti-MBP antibody. The same protein gel was used for a western blot involving the anti-GST antibody to verify that equal amounts of GST-PeaT1 were present in all protein mixtures. ^∗^ Represents the target protein band.

In order to excavate the molecular mechnism for *PeaT1*’s role in drought, a yeast two-hybrid screen using the Nipponbare cDNA was conducted to determine if there are any rice proteins which interact with PeaT1. We were also interested in characterizing the functions of any interacting protein. The BD-PeaT1 plasmid was used as the bait. Because PeaT1 contains a self-activated domain, we added 50 mM 3-AT to SD/-Leu/-Trp/-His medium. Dozens of potential interacting proteins were detected, including chloroplast precursor protein S1 and OsMIOX. To verify the accuracy of interaction, we made PeaT1 as the bait, and OsMIOX were as preys, respectively, in a yeast two-hybrid analysis. Finally, we found that there really existed interaction between PeaT1 and OsMIOX based on the results of X-gal (5-Bromo- 4-chloro-3-indolyl β-D-galactopyranoside) assays involving SD/-Leu/-Trp/-His medium supplemented with 3-AT (**Figure 3A**).

We also confirmed the interaction using a pull-down assay involving the PeaT1-MBP and OsMIOX1-GST fusion proteins (**Figure 3B**), which were produced in *Escherichia coli* cells. The OsMIOX1-GST fusion protein was able to pull down PeaT1-MBP, but GST alone could not (**Figure 3C**). These results verified the direct interaction between PeaT1 and OsMIOX.

### *OsMIOX* Expression Was Upregulated during the Drought Treatment

**FIGURE 4:**
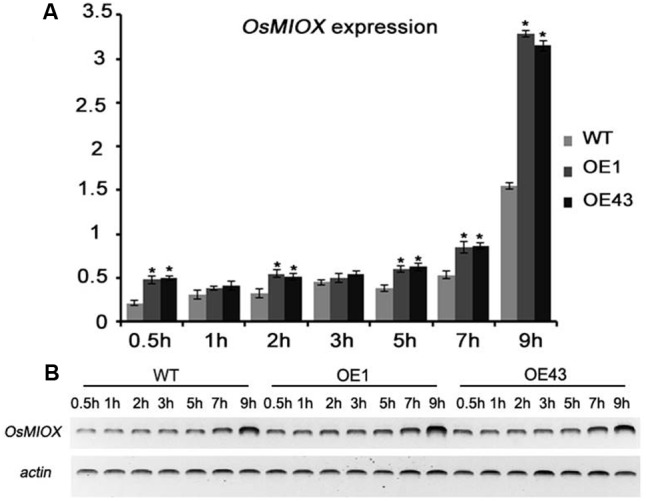
Expression of *OsMIOX* in WT and PeaT1OE plants exposed to air. **(A)** qRT-PCR analysis of *OsMIOX* expression. **(B)** *OsMIOX* expression was detected by semi-quantitative RT-PCR. Data is presented as the mean ± standard deviation (*n* = 9). Significant differences are determined with Student’s *t*-test (^∗^0.01 < *P* < 0.05, ^∗∗^*P* < 0.01).

To assess whether *OsMIOX* expression in PeaT1OE and WT plants was affected by drought stress, water-cultivated seedlings were exposed to air to simulate drought conditions. Leaves were collected at specific time points to measure the *OsMIOX* expression levels by qRT-PCR and semi-quantitative RT-PCR (**Figure 4**). The upregulated expression of *OsMIOX* followed a two-phase curve reminiscent of the interaction process. The first significant increase in PeaT1OE plants occurred at 0.5 and 2 h after the initiation of the drought treatment. A second peak was observed when PeaT1 interacted with OsMIOX at 5, 7, and 9 h after starting the drought treatment. There were no differences between the PeaT1OE and WT plants at the 1- and 3-h time points. These results suggested that PeaT1 might function cooperatively with OsMIOX during stress responses in PeaT1OE plants.

### Subcellular Localization of OsMIOX

**FIGURE 5:**
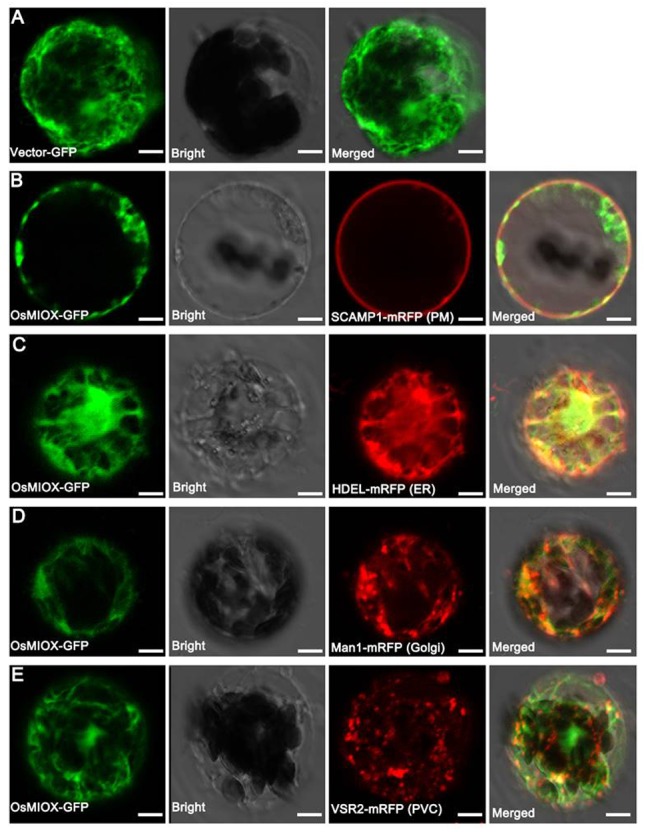
Subcellular localization of OsMIOX in protoplasts of *N. benthamiana* leaves. **(A)** Fluorescence signal from the expression of the GFP tag. Fluorescence signal from the co-expression of the OsMIOX-GFP fusion protein and **(B)** SCAMP1-mRFP (PM marker), **(C)** HDEL-mRFP (ER marker), **(D)** Man1-mRFP (Golgi marker), **(E)** VSR2-mRFP (PVC marker). The scale bar corresponds to **(A)** 9 μm, **(B)** 5 μm, **(C)** 8 μm, and **(D,E)** 10 μm. PM, plasma membrane; ER, endoplasmic reticulum; PVC, prevacuolar compartment.

To investigate the subcellular localization of OsMIOX, the *OsMIOX* cDNA was fused in frame to the GFP marker gene under the control of the cauliflower mosaic virus 35S promoter (35S::MIOX::GFP). The vector with the GFP gene was used as the control (**Figure 5A**). Mcherry fluorescent protein (mRFP)-tagged organelle markers (**Figures 5B–E**) were incorporated into the epidermal cells of *N. benthamiana* leaves. Protoplasts were prepared from the tobacco leaves to enhance the fluorescent signals detected by confocal microscopy. The MIOX-GFP fluorescence did not completely merge with the plasma membrane (PM: SCAMP1 [Lam et al., 2007]) (**Figure 5B**), which was consistent with previous results of predicted localization in cytoplasm. Furthermore, four organelle markers tagged with mRFP (Gomord et al., 1997; Nebenführ et al., 1999; Miao et al., 2006; Lam et al., 2007) were co-expressed with MIOX-GFP in tobacco protoplasts. We observed that the MIOX-GFP fluorescence co-localized only with the endoplasmic reticulum (ER) marker HDEL (**Figure 5C**).

## Discussion

Drought results in considerable adverse consequences for crop yield and quality. Previous studies have concluded that the PeaT1 elicitor is critical for the drought resistance and development in rice, wheat, and cotton plants (Liu et al., 2009; Wang et al., 2011; Tang et al., 2012). In the study, we observed that PeaT1OE plants showed increased drought-tolerant compared with WT, with higher survival rate in OE (**Figure 1**). Thus, PeaT1’s function in drought resistance is similar to another elicitor Harpin, which could enhances drought tolerance in rice (Zhang W. et al., 2011). These results suggested that PeaT1 played crucial roles in plant improvement tolerance of drought.

Expression of several genes related to drought resistance also changed differently between PeaT1OE plants and WT. For example, *OsDST* transduction significantly decreased in OE plants compared with WT (**Figure 2C**). As *OsDST* is a negative regulator in drought (Huang et al., 2009), lower expression of the gene might partially increase stomatal closure and reduce stomatal density, thus causing enhanced drought in OE plants. A study reported that the expression of *OsLP2* is down-regulated by drought and the gene’s overexpression plants are drought sensitivity (Wu et al., 2014). Our data detected that lower *OsLP2* expression existed in *PeaT1* OE plants than in the WT (**Figure 2B**), suggesting lower expression of *OsLP2* contributed to the plant drought resistance in OE. Taking together, these results implied that the overexpression of *PeaT1* in plants might impact the transduction of other drought-responsive genes, thereby inducing OE plants drought tolerance.

Abscisic acid exerts key roles in affecting stomatal defense against drought stresses via activating diverse plant physiological and developmental processes (Finkelstein et al., 2002; Robertseilaniantz et al., 2007; Ton et al., 2009). Under drought conditions, ABA is produced or accumulated to induce stomatal closure, ultimately helping plant conserve water (Bauer et al., 2013; Lee and Luan, 2012). ABA-controlled processes are necessary for plant survival and deficient mutants generally are susceptible to water stress (Finkelstein et al., 2002; Kang et al., 2002). The upregulated expression of *NCED3* (an ABA biosynthesis gene) is induced by drought stress, in turn decreasing transpiration rates and enhancing plant water stress (Schwartz et al., 1997, 2003; Tan et al., 1997; Thompson et al., 2000). Here, we analyzed the expression of *OsNCED2* (another ABA biosynthesis gene) and found that it was significantly increased in PeaT1OE plants (**Figure 2F**), similar to *NCED3*’s upregulation, which both improve plant drought resistance. These results indicated that overexpressing *PeaT1* affected some ABA biosynthesis genes expression, which increased ABA content, finally improved plant drought tolerance. However, several ABA signaling genes expression should be detected in future in order to know whether upregulation of *PeaT1* has effects on ABA signal tranduction.

Myo-inositol oxygenase (MIOX) could regulate the abundance of *myo*-inositol, thus affecting the ascorbic acid biosynthesis (Kanter et al., 2005). Ascorbic acid, as a major plant antioxidant, plays roles in countering balances of any oxidative damage (Shao et al., 2008). However, little knowledge about the sub-location of MIOX, except rice OsMIOX was predicted to be localized in cytoplasm (Duan et al., 2012), it was unclear whether it is associated with specific subcellular structures. In the study, we co-expressed OsMIOX with crucial organelle markers in tobacco protoplasts and observed that OsMIOX just co-localized with ER marker, suggesting OsMIOX functions in ER.

A recent study showed that overexpression of *OsMIOX* in rice could improve drought tolerance (Duan et al., 2012). Our results showed that *OsMIOX* expression was significantly upregulated in PeaT1OE plants under drought conditions, suggesting that *PeaT1* might promote the transcription of *OsMIOX*. However, the molecuar function of *PeaT1* or *OsMIOX* on drought tolerance remians unclear. By yeast screening system, we found that PeaT1 could interact with OsMIOX *in vivo* and *in vitro*, which offered evidence of PeaT1’s roles in drought reistance on molecular level. And it is the first report that elicitor PeaT1 could physicall interact with Myo-inositol oxygenase together to carry out their function. Further studies on whether PeaT1 interacting with other protein will be required to deeply understand PeaT1’s molecular function.

## Author Contributions

XY and DQ designed experiments. FS carried out experiments and analyzed experimental results. FS wrote the manuscript. YZ and YD assistant the experiment and wrote the MS.

## Conflict of Interest Statement

The authors declare that the research was conducted in the absence of any commercial or financial relationships that could be construed as a potential conflict of interest.

## References

[B1] Ahmad I. Devonshire J. Mohamed R. Chultze M. Maathuis F. J. (2016). Overexpression of the potassium channel TPKb in small vacuoles confers osmotic and drought tolerance to rice. *New Phytol.* 209 1040–1048. 10.1111/nph.13708 26474307

[B2] Antoni R. Rodriguez L. Gonzalez-Guzman M. Pizzio G. A. Rodriguez P. L. (2011). News on ABA transport, protein degradation, and ABFs/WRKYs in ABA signaling. *Curr. Opin. Plant Biol.* 14 547–553. 10.1016/j.pbi.2011.06.004 21742545

[B3] Bates L. S. Waldren R. P. Teare I. D. (1973). Rapid determination of free proline for water-stress studies. *Plant Soil* 39 205–207. 10.1016/j.dental.2010.07.006

[B4] Batoko H. Zheng H. Q. Hawes C. Moore I. (2000). A Rab1 GTPase is required for transport between the endoplasmic reticulum and Golgi apparatus and for normal Golgi movement in plants. *Plant Cell* 12 2201–2218. 10.1105/tpc.12.11.2201 11090219 PMC150168

[B5] Bauer H. Ache P. Lautner S. Fromm J. Hartung W. Alrasheid K. A. S. (2013). The stomatal response to reduced relative humidity requires guard cell-autonomous ABA synthesis. *Curr. Biol.* 23 53–57. 10.1016/j.cub.2012.11.022 23219726

[B6] Duan J. Zhang M. Zhang H. Xiong H. Liu P. Ali J. (2012). *OsMIOX*, a *myo*-inositol oxygenase gene, improves drought tolerance through scavenging of reactive oxygen species in rice (*Oryza sativa* L.). *Plant Sci.* 196 143–151. 10.1016/j.plantsci.2012.08.003 23017909

[B7] Fan L. M. Zhao Z. Assmann S. M. (2004). Guard cells: a dynamic signaling model. *Curr. Opin. Plant Biol.* 7 537–546. 10.1016/j.pbi.2004.07.009 15337096

[B8] Finkelstein R. R. Gampala S. S. L. Rock C. D. (2002). Abscisic acid signaling in seeds and seedlings. *Plant Cell* 14 S15–S45. 12045268 10.1105/tpc.010441 PMC151246

[B9] Fu J. Chu J. Sun X. Wang J. Yan C. (2012). Simple, rapid, and simultaneous assay of multiple carboxyl containing phytohormones in wounded tomatoes by UPLC-MS/MS using single SPE purification and isotope dilution. *Anal. Sci.* 28 1081–1087. 10.2116/analsci.28.1081 23149609

[B10] Fujita Y. Fujita M. Shinozaki K. Yamaguchishinozaki K. (2011). ABA-mediated transcriptional regulation in response to osmotic stress in plants. *J. Plant Res.* 124 509–525. 10.1007/s10265-011-0412-3 21416314

[B11] Gomord V. Denmat L. A. Fitchette-Lainé A. C. Satiat-Jeunemaitre B. Hawes C. Faye L. (1997). The C-terminal HDEL sequence is sufficient for retention of secretory proteins in the endoplasmic reticulum (ER) but promotes vacuolar targeting of proteins that escape the ER. *Plant J.* 11 313–325. 10.1046/j.1365-313X.1997.11020313.x 9076996

[B12] Gonzalez-Guzman M. Pizzio G. A. Antoni R. Vera-Sirera F. Merilo E. Bassel G. W. (2012). Arabidopsis PYR/PYL/RCAR receptors play a major role in quantitative regulation of stomatal aperture and transcriptional response to abscisic acid. *Plant Cell* 24 2483–2496. 10.1105/tpc.112.098574 22739828 PMC3406898

[B13] He S. Y. Huang H. C. Collmer A. (1993). *Pseudomonas syringae* pv. *syringae* harpin_Pss_: a protein that is secreted via the Hrp pathway and elicits the hypersensitive response in plants. *Cell* 73 1255–1266. 10.1016/0092-8674(93)90354-S 8324821

[B14] Heath R. L. Packer L. (1968). Photoperoxidation in isolated chloroplasts: I. Kinetics and stoichiometry of fatty acid peroxidation. *Arch. Biochem. Biophys.* 125 189–198. 10.1016/0003-9861(68)90654-1 5655425

[B15] Huang X. Y. Chao D. Y. Gao J. P. Zhu M. Z. Shi M. Lin H. X. (2009). A previously unknown zinc finger protein, DST, regulates drought and salt tolerance in rice via stomatal aperture control. *Genes Dev.* 23 1805–1817. 10.1101/gad.1812409 19651988 PMC2720257

[B16] Jiang Y. Qiu Y. Hu Y. Yu D. (2016). Heterologous expression of AtWRKY57 confers drought tolerance in *Oryza sativa*. *Front. Plant Sci.* 7:145 10.3389/fpls.2016.00145 PMC4749717 26904091

[B17] Jisha V. Dampanaboina L. Vadassery J. Mithöfer A. Kappara S. Ramanan R. (2015). Overexpression of an AP2/ERF type transcription factor *OsEREBP1* confers biotic and abiotic stress tolerance in rice. *PLoS ONE* 10:e0127831 10.1371/journal.pone.0127831 PMC4452794 26035591

[B18] Kang J. Y. Choi H. I. Im M. Y. Kim S. Y. (2002). Arabidopsis basic leucine zipper proteins that mediate stress-responsive abscisic acid signaling. *Plant Cell* 14 343–357. 10.1105/tpc.010362 11884679 PMC152917

[B19] Kanter U. Usadel B. Guerineau F. Li Y. Pauly M. (2005). The inositol oxygenase gene family of *Arabidopsis* is involved in the biosynthesis of nucleotide sugar precursors for cell-wall matrix polysaccharides. *Planta* 221 243–254. 10.1007/s00425-004-1441-0 15660207

[B20] Kim T. H. (2014). Mechanism of ABA signal transduction: agricultural highlights for improving drought tolerance. *J. Plant Biol.* 57 1–8. 10.1007/s12374-014-0901-8

[B21] Kong Z. Li M. Yang W. Xu W. Xue Y. (2006). A novel nuclear-localized CCCH-type zinc finger protein, OsDOS, is involved in delaying leaf senescence in rice. *Plant Physiol.* 141 1376–1388. 10.1104/pp.106.082941 16778011 PMC1533915

[B22] Kwak J. M. Mori I. C. Pei Z. M. Leonhardt N. Torres M. A. Dangl J. L. (2003). NADPH oxidase *AtrbohD* and *AtrbohF* genes function in ROS-dependent ABA signaling in *Arabidopsis*. *EMBO J.* 22 2623–2633. 10.1093/emboj/cdg277 12773379 PMC156772

[B23] Lam S. K. Siu C. L. Hillmer S. Jang S. An G. Robinson D. G. (2007). Rice SCAMP1 defines clathrin-coated, *trans*-golgi–located tubular-vesicular structures as an early endosome in tobacco BY-2 cells. *Plant Cell* 19 296–319. 10.1105/tpc.106.045708 17209124 PMC1820953

[B24] Lee S. C. Lim C. W. Lan W. He K. Luan S. (2013). ABA signaling in guard cells entails a dynamic protein–protein interaction relay from the PYL-RCAR family receptors to ion channels. *Mol. Plant* 6 528–538. 10.1093/mp/sss078 22935148

[B25] Lee S. C. Luan S. (2012). ABA signal transduction at the crossroad of biotic and abiotic stress responses. *Plant Cell Environ.* 35 53–60. 10.1111/j.1365-3040.2011.02426.x 21923759

[B26] Li G. L. Wu H. X. Sun Y. Q. Zhang S. Y. (2013). Response of chlorophyll fluorescence parameters to drought stress in sugar beet seedlings. *Russ. J. Plant Physiol.* 60 337–342. 10.1134/S1021443713020155

[B27] Lim C. W. Baek W. Jung J. Kim J. H. Lee S. C. (2015). Function of ABA in stomatal defense against biotic and drought stresses. *Int. J. Mol. Sci.* 16 15251–15270. 10.3390/ijms160715251 26154766 PMC4519898

[B28] Liu Q. Guang-Yue L. I. Zeng H. M. Yang X. F. Qiu D. W. (2009). Acquisition of microbial protein elicitor PeaT1 and preliminary research on inducing drought resistance of rice. *J. Agric. Sci. Technol.* 11 51–55.

[B29] Livak K. J. Schmittgen T. D. (2001). Analysis of relative gene expression data using real-time quantitative pcr and the 2^-ΔΔC_T_^ method. *Methods* 25 402–408. 10.1006/meth.2001.1262 11846609

[B30] Ma Y. Szostkiewicz I. Korte A. Moes D. Yang Y. Christmann A. (2009). Regulators of PP2C phosphatase activity function as abscisic acid sensors. *Sci.* 324 1064–1068. 10.1126/science.1172408 19407143

[B31] Marone M. Mozzetti S. De R. D. Pierelli L. Scambi G. (2001). Semiquantitative RT-PCR analysis to assess the expression levels of multiple transcripts from the same sample. *Biol. Proced. Online* 3 19–25. 10.1251/bpo20 12734582 PMC145543

[B32] Miao Y. Yan P. K. Kim H. Hwang I. Jiang L. (2006). Localization of green fluorescent protein fusions with the seven Arabidopsis vacuolar sorting receptors to prevacuolar compartments in tobacco BY-2 cells. *Plant Physiol.* 142 945–962. 10.1104/pp.106.083618 16980567 PMC1630755

[B33] Nebenführ A. Gallagher L. A. Dunahay T. G. Frohlick J. A. Mazurkiewicz A. M. Meehl J. B. (1999). Stop-and-go movements of plant Golgi stacks are mediated by the acto-myosin system. *Plant Physiol.* 121 1127–1141. 10.1104/pp.121.4.1127 10594100 PMC59480

[B34] Nishimura N. Sarkeshik A. Nito K. Park S. Y. Wang A. Carvalho P. C. (2009). PYR/PYL/RCAR family members are major *in-vivo* ABI1 protein phosphatase 2C-interacting proteins in Arabidopsis. *Plant J.* 61 290–299. 10.1111/j.1365-313X.2009.04054.x 19874541 PMC2807913

[B35] Nürnberger T. (1999). Signal perception in plant pathogen defense. *Cell. Mol. Life Sci. CMLS* 55 167–182. 10.1007/s000180050283 24481912 PMC11146998

[B36] Paré P. W. Farag M. A. Krishnamachari V. Zhang H. Ryu C. M. Kloepper J. W. (2005). Elicitors and priming agents initiate plant defense responses. *Photosynth. Res.* 85 149–159. 10.1007/s11120-005-1001-x 16075316

[B37] Park S. Y. Fung P. Nishimura N. Jensen D. R. Fujii H. Zhao Y. (2009). Abscisic acid inhibits type 2C protein phosphatases via the PYR/PYL family of START proteins. *Science* 324 1068–1071. 10.1126/science.1173041 19407142 PMC2827199

[B38] Pei Z. M. Murata Y. Benning G. Thomine S. Klüsener B. Allen G. J. (2000). Calcium channels activated by hydrogen peroxide mediate abscisic acid signalling in guard cells. *Nature* 406 731–734. 10.1038/35021067 10963598

[B39] Redillas M. C. Jeong J. S. Kim Y. S. Jung H. Bang S. W. Choi Y. D. (2012). The overexpression of OsNAC9 alters the root architecture of rice plants enhancing drought resistance and grain yield under field conditions. *Plant Biotechnol. J.* 10 792–805. 10.1111/j.1467-7652.2012.00697.x 22551450

[B40] Robertseilaniantz A. Navarro L. Bari R. Jones J. D. (2007). Pathological hormone imbalances. *Curr. Opin. Plant Biol.* 10 372–379. 10.1016/j.pbi.2007.06.003 17646123

[B41] Schwartz S. H. Qin X. Zeevaart J. A. (2003). Elucidation of the indirect pathway of abscisic acid biosynthesis by mutants, genes, and enzymes. *Plant Physiol.* 131 1591–1601. 10.1104/pp.102.017921 12692318 PMC1540303

[B42] Schwartz S. H. Tan B. C. Gage D. A. Zeevaart J. A. D. Mccarty D. R. (1997). Specific oxidative cleavage of carotenoids by VP14 of maize. *Science* 276 1872–1874. 10.1126/science.276.5320.1872 9188535

[B43] Seki M. Umezawa T. Urano K. Shinozaki K. (2007). Regulatory metabolic networks in drought stress responses. *Curr. Opin. Plant Biol.* 10 296–302. 10.1016/j.pbi.2007.04.014 17468040

[B44] Shao H. B. Chu L. Y. Shao M. A. Jaleel C. A. Mi H. M. (2008). Higher plant antioxidants and redox signaling under environmental stresses. *C. R. Biol.* 331 433–441. 10.1016/j.crvi.2008.03.011 18510996

[B45] Sheng D. Yushanjiang M. Guo L. Zeng H. Yang X. Yuan J. (2011). Transfer of the protein elicitor gene peaT1 into duoxi 1. *Mol. Plant Breed.* 9 1450–1456.

[B46] Sheng P. Tan J. Jin M. Wu F. Zhou K. Ma W. (2014). Albino midrib 1, encoding a putative potassium efflux antiporter, affects chloroplast development and drought tolerance in rice. *Plant Cell Rep.* 33 1581–1594. 10.1007/s00299-014-1639-y 24917171

[B47] Smirnoff N. Conklin P. L. Loewus F. A. (2001). BIOSYNTHESIS OF ASCORBIC ACID IN PLANTS: a renaissance. *Plant Biol.* 52 437–467. 10.1146/annurev.arplant.52.1.437 11337405

[B48] Tan B. C. Schwartz S. H. Zeevaart J. A. D. Mccarty D. R. (1997). Genetic control of abscisic acid biosynthesis in maize. *Proc. Natl. Acad. Sci. U.S.A.* 94 12235–12240. 10.1073/pnas.94.22.12235 9342392 PMC23760

[B49] Tang H. Zeng H. Yang X. Li G. Sheng D. Yuan J. (2012). Identification of transgenic cotton with a protein elicitor-encoding gene peaT1. *Biotechnol. Bull.* 1 60–64.

[B50] Thompson A. J. Jackson A. C. Symonds R. C. Mulholland B. J. Dadswell A. R. Blake P. S. (2000). Ectopic expression of a tomato 9-cis-epoxycarotenoid dioxygenase gene causes over-production of abscisic acid. *Plant J.* 23 363–374. 10.1046/j.1365-313x.2000.00789.x 10929129

[B51] Ton J. Flors V. Mauch-Mani B. (2009). The multifaceted role of ABA in disease resistance. *Trends Plant Sci.* 14 310–317. 10.1016/j.tplants.2009.03.006 19443266

[B52] Wang L. Yang X. Zeng H. Qiu D. Guo L. Liu Z. (2011). Extracellular expression of protein elicitor PeaT1 in *Bacillus subtilis* to enhance drought tolerance and growth in wheat. *Chin. J. Biotechnol.* 27 1355–1362. 22117519

[B53] Wei S. Hu W. Deng X. Zhang Y. Liu X. Zhao X. (2014). A rice calcium-dependent protein kinase OsCPK9 positively regulates drought stress tolerance and spikelet fertility. *BMC Plant Biol.* 14:133 10.1186/1471-2229-14-133 PMC4036088 24884869

[B54] Wei Z. M. Laby R. J. Zumoff C. H. Bauer D. W. He S. Y. Collmer A. (1992). Harpin, elicitor of the hypersensitive response produced by the plant pathogen *Erwinia amylovora*. *Science* 257 85–88. 10.1126/science.1621099 1621099

[B55] Wu F. Sheng P. Tan J. Chen X. Lu G. Ma W. (2014). Plasma membrane receptor-like kinase leaf panicle 2 acts downstream of the DROUGHT AND SALT TOLERANCE transcription factor to regulate drought sensitivity in rice. *J. Exp. Bot.* 66 271–281. 10.1093/jxb/eru417 25385766 PMC4265162

[B56] Zhang L. Xiao S. Li W. Feng W. Li J. Wu Z. (2011). Overexpression of a Harpin-encoding gene *hrf1* in rice enhances drought tolerance. *J. Exp. Bot.* 62 4229–4238. 10.1093/jxb/err131 21527628 PMC3153678

[B57] Zhang W. Yang X. Qiu D. Guo L. Zeng H. (2011). PeaT1-induced systemic acquired resistance in tobacco follows salicylic acid-dependent pathway. *Mol. Biol. Rep.* 38 2549–2556. 10.1007/s11033-010-0393-7 21088909

[B58] Zhang Y. Yang X. Liu Q. Qiu D. Zhang Y. Zeng H. (2010). Purification of novel protein elicitor from *Botrytis cinerea* that induces disease resistance and drought tolerance in plants. *Microbiol. Res.* 165 142–151. 10.1016/j.micres.2009.03.004 19616421

[B59] Zhou F. Lin Q. Zhu L. Ren Y. Zhou K. Shabek N. (2013). D14-SCF^D3^-dependent degradation of D53 regulates strigolactone signalling. *Nature* 504 406–410. 10.1038/nature12878 24336215 PMC4096652

[B60] Zhu J. K. (2002). Salt and drought stress signal transduction in plants. *Annu. Rev. Plant Biol.* 53 247–273. 10.1146/annurev.arplant.53.091401.143329 12221975 PMC3128348

