# Supplementary material for: Overexpression of the PeaT1 Elicitor Gene from Alternaria tenuissima Improves Drought Tolerance in Rice Plants via Interaction with a Myo-Inositol Oxygenase
Source: Front Plant Sci. 2017 Jun 9;8:970. doi: 10.3389/fpls.2017.00970 (PMC5465376; doi:10.3389/fpls.2017.00970)
Supplement: Supplementary file 1 [file Table_1.docx]

Table S1. List of primers

| **Primers** | **Forward sequence (5'- 3')** | **Reverse sequence (5'- 3')** |
| --- | --- | --- |
| ***PeaT1-BD*** | AGGTCGAC ATGGCCAACCCCCGCATTGA | CTTAGCGGCCGC TATGCTCAGCGCCATGATGGA |
| ***OsMIOX-AD*** | AAATCGACCGAGGGTGGGATGACCATCACCATTGAGC | GATCTGAATTCCAGCTGGCCATCTCAACTTGGCC |
| ***PeaT1-eYNE*** | CGCCACTAGTGGATCCATGGCCAACC CCCGCATTGA AGAG | TACCCTCGAGGTCGACTATGCTCAGCGCCATGATGGA |
| ***OsMIOX-eYCE*** | CGCCACTAGTGGATCCATGACCATCACCATTGAGCAGCCTCA | TACCCTCGAGGTCGAC CCATCTCAACTTGGCCGGGAAATA |
| ***OsMIOX–MBP*** | AAGGATTTCAGAATTCATGACCATCACCATTGAGCAGCCTCA | TAGAGGATCCGAATTCCATCTCAACTTGGCCGGGAAATA |
| ***PeaT1- GST*** | TGGGTCGCGGATCCGAAATGGCCAACCCCCGCATTGAAGA | GACGGAGCTCGAATT TATGCTCAGCGCCATGATGGAGT |
| ***OsMIOX-1305GFP*** | CGAGCTGTACAGATCTATGACCATCACCATTGAGCAGCCTCA | GGCCGCTTTAAGATCTCCATCTCAACTTGGCCGGGAAATA |
| ***OsAM1*** | GGCTGTGGTGGTCCTGTTGA | ACAGACGACCTCCAGCGATTAT |
| ***OsLP2*** | ATGGGCCTCACGTGTGATAC | CAAGAAGGGCATCACCAACG |
| ***OsDST*** | ATCCAAGAAGGCAAGGTCAATC | ACACACGAGGAGGAATTGGAA |
| ***OsNCED2*** | TCCGTTGCCCAAGATCAAG | CGTCCAACCGTGCAATCAC |
| ***OsMIOX*** | TCATTCATTCTACCCCCTGC | GGCTTCACCTTCTCAACGTC |
| ***UBQ*** | ACCCTGGCTGACTACAACATC | AGTTGACAGCCCTAGGGTG |
| ***OsCPK9*** | TTGACGGCAGCGGCTACAT | GCTGTTGCTGAGCGTCTTG |
| ***OsNAC9*** | CTGAGCTACGACGATATCCA | GAAGAGCGACGAGTAGAAGT |
| ***OsTPKb*** | GCTGCACTCGCACACGAT | CCCCGCCGTGTAGAGCTT |
| ***OsEREBP1*** | ACTGCCGGATTTGATGGTCCTG | CAGCATCATAAGCTCTTGCAGC |
| ***Osactin*** | ATGGCTGACGCCGAGGATATCC | TTAGAAGCATTTCCTGTGCACA |
| ***MIOX*** | ATGACCATCACCATTGAGCA | TCACCATCTCAACTTGGCCGGG |
| ***OsSKIPa*** | CGAAATTCAAGCATAAGCGA | TGAAATGCATGGTGGAATCT |
